# Discovery of Novel dsRNA Viral Sequences by *In Silico* Cloning and Implications for Viral Diversity, Host Range and Evolution

**DOI:** 10.1371/journal.pone.0042147

**Published:** 2012-07-27

**Authors:** Huiquan Liu, Yanping Fu, Jiatao Xie, Jiasen Cheng, Said A. Ghabrial, Guoqing Li, Xianhong Yi, Daohong Jiang

**Affiliations:** 1 State Key Laboratory of Agricultural Microbiology, Huazhong Agricultural University, Wuhan, Hubei Province, People's Republic of China; 2 The Provincial Key Lab of Plant Pathology of Hubei Province, College of Plant Science and Technology, Huazhong Agricultural University, Wuhan, Hubei Province, People's Republic of China; 3 Department of Plant Pathology, University of Kentucky, Lexington, Kentucky, United States of America; Natural History Museum of Denmark, University of Copenhagen, Denmark

## Abstract

Genome sequence of viruses can contribute greatly to the study of viral evolution, diversity and the interaction between viruses and hosts. Traditional molecular cloning methods for obtaining RNA viral genomes are time-consuming and often difficult because many viruses occur in extremely low titers. DsRNA viruses in the families, *Partitiviridae*, *Totiviridae, Endornaviridae*, *Chrysoviridae*, and other related unclassified dsRNA viruses are generally associated with symptomless or persistent infections of their hosts. These characteristics indicate that samples or materials derived from eukaryotic organisms used to construct cDNA libraries and EST sequencing might carry these viruses, which were not easily detected by the researchers. Therefore, the EST databases may include numerous unknown viral sequences. In this study, we performed *in silico* cloning, a procedure for obtaining full or partial cDNA sequence of a gene by bioinformatics analysis, using known dsRNA viral sequences as queries to search against NCBI Expressed Sequence Tag (EST) database. From this analysis, we obtained 119 novel virus-like sequences related to members of the families, *Endornaviridae*, *Chrysoviridae*, *Partitiviridae*, and *Totiviridae*. Many of them were identified in cDNA libraries of eukaryotic lineages, which were not known to be hosts for these viruses. Furthermore, comprehensive phylogenetic analysis of these newly discovered virus-like sequences with known dsRNA viruses revealed that these dsRNA viruses may have co-evolved with respective host supergroups over a long evolutionary time while potential horizontal transmissions of viruses between different host supergroups also is possible. We also found that some of the plant partitiviruses may have originated from fungal viruses by horizontal transmissions. These findings extend our knowledge of the diversity and possible host range of dsRNA viruses and offer insight into the origin and evolution of relevant viruses with their hosts.

## Introduction

The genome sequence of viruses can contribute greatly to the study of viral evolution, diversity and interactions between viruses and their hosts. Traditional methods for obtaining RNA viral sequences include the use of techniques such as dsRNA isolation, cDNA library construction and molecular cloning [1], [2]. Though powerful in discovering unknown viruses, these methods are time-consuming and often hindered by difficulties in cultivation and extremely low titers for many viruses. Amplification by PCR based on known viral sequences is most efficient for detecting viruses, but only leads to discovery of known or similar viruses. Recent viral metagenomic studies overcame these limitations and provided a promising method for investigation of unrefined viral diversity, in which viral particles are first partially purified and then viral sequences are randomly amplified before sub-cloning and sequencing [3], [4]. By using this approach, numerous previously unknown viruses have been discovered in environmental and clinical samples [5], [6], [7], [8]. However, a disadvantage of this method is difficulties to identify the host range of detected viruses. With the advance of next generation sequencing (NGS) technologies, another culture-independent approach for virus discovery is developed by deep sequencing and assembly of virus-derived small silencing RNAs [9], [10], [11]. This approach can identify both plant and invertebrate viruses occurring at extremely low titers without purification of viral particles and amplification of viral sequences.

Double-stranded (ds) viruses infecting eukaryotes are grouped into seven families: *Birnaviridae*, *Picobirnaviridae*, *Reoviridae, Endornaviridae*, *Chrysoviridae*, *Partitiviridae*, and *Totiviridae* [12]. Though many members in the first three families cause serious diseases, viruses in the latter four families are generally associated with latent infections and have little or no overt effects on their hosts. Members of family *Totiviridae* possess a monopartite genome encoding, in most cases, only a capsid protein (CP) and an RNA-dependent RNA polymerase (RdRp) [12], [13]. These viruses mainly infect fungi and protozoa. Viruses in the family *Partitiviridae* infect fungi, plants or apicomplexan protozoa and possess a bipartite genome separately encoding the CP and RdRp [12], [13], [14], [15], [16]. The family *Chrysoviridae* encompasses viruses with quadripartite genomes that code for RdRp, CP, and two unknown proteins P3 and P4 [12], [13], [17]. Currently, the known host range of chrysoviruses is limited to fungi. Members of family *Endornaviridae* comprise large dsRNAs encoding a single long polypeptide with typical viral RNA helicases (Hels), UDP-glucosyltransferases (UGTs), and RdRps [18]. Endornaviruses have been reported in plants, fungi, and oomycetes. Recently, several monopartite dsRNA viruses distantly related to totiviruses and partitiviruses were found in plants [19], [20], [21]. In addition, a novel bipartite dsRNA virus and a novel quadripartite dsRNA virus phylogenetically related to chrysoviruses and totiviruses were reported in fungi [22], [23]. These viruses may belong to novel dsRNA viral families [19], [22], [23]. Hence, our understanding of the diversity and host range of dsRNA viruses must rely on the discovery of additional new viruses.

The partitiviruses, totiviruses, chrysoviruses, and endornaviruses as well as other related unclassified dsRNA viruses do not have extracellular routes for infection and are transmitted vertically via cell division or horizontally via cell fusions. Therefore, it has been suggested that these viruses may have co-evolved with their hosts [24], [25]. However, Koonin and co-authors [26] suggested that horizontal transmission of viruses between plants and fungi (interkingdom host jumping) might have been particularly important in the evolution of the family *Partitiviridae*. Indeed, recent phylogenetic analyses based on amino acid sequences of RdRps of partitiviruses suggest that partitiviral horizontal transmission between fungi and plants may have occurred [15]. However, due to limited availability of genomic sequences of representative viruses, evolutionary relationships of these viruses with their hosts remain to be elucidated.

Considering that many dsRNA viruses are associated with symptomless or persistent infections of their hosts, samples or materials derived from eukaryotic organisms used to construct cDNA libraries for EST sequencing may carry viruses. Viral RNAs may have been cloned and sequenced together with host RNAs. Therefore, the EST databases may include numerous viral ESTs that are treated as contaminating sequences. These viral ESTs, however, are important to understand the host range and evolution of dsRNA viruses. With this in mind, we performed *in silico* cloning, a procedure of obtaining full or partial cDNA sequence of a gene by bioinformatics analysis, using the known dsRNA viral sequences as queries to search against NCBI Expressed Sequence Tag (EST) database. In this study, we obtained numerous virus-like sequences related to members of families *Endornaviridae*, *Chrysoviridae*, *Partitiviridae*, and *Totiviridae*. Many of them were discovered from eukaryotic lineages that were not known to be hosts to these viruses. Furthermore, we conducted comprehensive phylogenetic analysis with these newly identified virus-like sequences and known dsRNA viruses. Results from this study extended our knowledge of the diversity and possible host range of dsRNA viruses and offered insight into the origin and evolution of relevant viruses with their hosts.

## Results and Discussion

### Identification of novel partitivirus-like sequences

**Figure 1:**
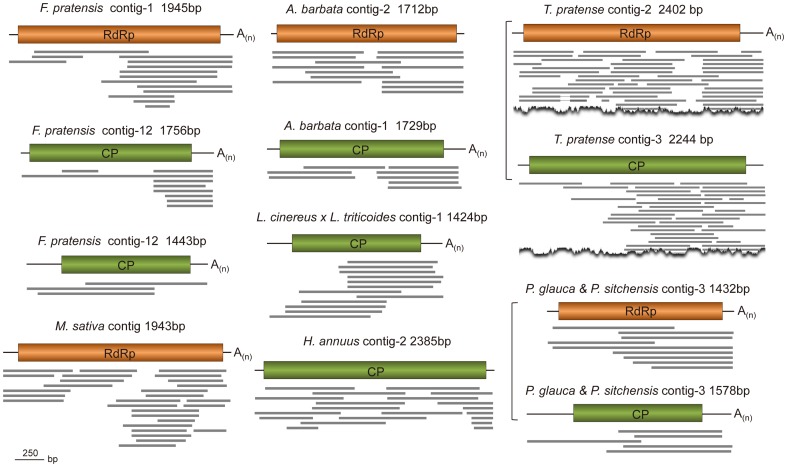
Representative full-length sequences of partitiviral RdRp or CP segments assembled from EST libraries of different plants. Multiple ESTs were used to construct contigs where overlapping regions of EST sequences show over 97% sequence identity. Bracket indicates that the RdRp and CP-like sequences were possibly from one virus. Rectangular boxes indicate ORFs.

By using the *in silico* cloning method, we obtained 91 virus-like sequences (contigs or singletons) that were most closely related to members of the family *Partitiviridae* (Table 1 and Data S1). Among these partitivirus-like sequences, 47 were RdRp-like and 44 were CP-like sequences. Despite the fact that most of them represented only partitiviral genomic fragments, some assembled contigs contains complete or near full-length sequence of RdRp or CP (Fig. 1). Most of the partitivirus-like sequences (18) were discovered from plant cDNA libraries, including those of 10 monocots, 19 eudicots, and 3 conifers, but only two were found in fungal cDNA libraries, although these viruses are common in different fungal species. It is possible that plant materials carrying partitiviruses used to construct cDNA libraries render viral detection more difficult than fungal materials. In fact, the concentration of partitiviruses in plants was often low while it was relatively high in fungi. On the other hand, the currently available fungal EST data are limited.

**Table 1:** Partitivirus-like sequences discovered from cDNA libraries of eukaryotic organisms.

Organism group	Organisma)	No. of RdRp		No. of CP		Totalc)
		Contigb)	Singleton	Contigb)	Singleton	
Plants						
monocots	*Asparagus officinalis* (garden asparagus)			1		1
	*Festuca arundinacea* (tall fescue)	1				1
	*Festuca pratensis* (meadow ryegrass)	6 (1)	4	3 (2)		13
	*Lolium perenne* (perennial ryegrass)			1		1
	*Lolium multiflorum* (Italian ryegrass)	1		2		3
	*Avena barbata* (slender oat)	1 (1)		1 (1)		2
	*Secale cereale* (rye)		1	1		2
	*Leymus cinereus x Leymus triticoides* (basin, creeping wild rye)	2		1 (1)		3
	*Pseudoroegneria spicata* (beardless wheatgrass)		2			3
	*Agrostis capillaris* (waipu)	1				1
eudicots	*Triphysaria versicolor* (Yellowbeak owl's-clover)			1		1
	*Triphysaria pusilla* (dwarf owl's clover)				2	2
	*Lactuca perennis* (blue lettuce)				1	1
	*Medicago sativa* (alfalfa)	1 (1)			2	2
	*Trifolium pratense* (red clover)	1 (1)		2 (1)		3
	*Quercus mongolica var. crispula* (Mongolian oak)			2	1	3
	*Eschscholzia californica* (California poppy)	1		1		2
	*Artemisia annua* (sweet wormwood)	1	2			3
	*Allium cepa* (onion)			1 (1)		1
	*Brassica rapa subsp. pekinensis* (Chinese cabbage)		1		2	3
	*Raphanus raphanistrum subsp. raphanistrum* (wild radish)	1 (1)		2	1	4
	*Raphanus raphanistrum subsp. maritimus* (Sea Radish)	1 (1)			4	5
	*Helianthus annuus* (common sunflower)	1 (1)		3		4
	*Helianthus paradoxus* (Puzzle Sunflower)	1				1
	*Gossypium hirsutum* (upland cotton)				1	1
	*Chenopodium glaucum* (oakleaf goosefoot)		1			1
	*Cannabis sativa* (hemp)	2		1 (1)		3
	*Cynara scolymus* (artichoke)		1			1
	*Salvia fruticosa* (Greek sage)		3			3
conifers	*Picea glauca & Picea sitchensis* (white spruce & Sitka spruce)*	1 (1)		2 (1)		3
	*Pseudotsuga menziesii var. menziesii* (Douglas-fir)		1			1
Fungi	*Conidiobolus coronatus*		1			1
	*Pisolithus microcarpus*	1				1
Animals	*Schistosoma mansoni* (flatworms)				1	1
	*Botrylloides leachi* (tunicates)		1			1
	*Ascaris suum* (pig roundworm)	1	1			2
	*Phlebotomus papatasi* (Sandfly)		1			1
	*Onychiurus arcticus* (Tullberg)	1				1
	*Mengenilla chobauti* (twisted-wing parasites)				1	1
	*Gadus morhua* (Atlantic cod)		1			1
Plants/Fungi	*Oryza sativa* (rice) infected by *Magnaporthe grisea*			1		1
	*Capsicum annuum* (Chili Pepper) infected by *Pytophthora capsici*			1	1	2
Total	43	26	21	27	17	91

a) Partitivirus-like ESTs were detected from the cDNA libraries of these organisms. Note the possibility that certain viral sequences are possible not from the annotated host organisms.

* ESTs from the two species are high identical and are assembled into one contig.

b) The numbers in parentheses indicate the numbers of contigs which are corresponding to complete or near full-length sequence of RdRp or CP segments. Note the possibility that virus ESTs used to generate a contig are possible not from the same virus.

c) Several sequences in certain species were possibly corresponding to one viral segment but were not assembled into one contig due to sequencing gaps.

Interestingly, though partitiviruses have not been isolated from animals thus far, we discovered 8 partitivirus-like sequences from the cDNA libraries of 7 animal species.

Most of these virus-like sequences shared low amino acid (aa) identities (<60%) with those of known partitiviruses, suggesting that they may represent new viral species in the *Partitiviridae* family. However, some of them, such as a few sequences from wild radish (*Raphanus raphanistrum subsp. raphanistrum*) and sea radish (*R. raphanistrum subsp. maritimus*) have high sequence identity (>90% aa) to RdRp or CP of *R. sativus* cryptic virus 2 (RsCV-2) and RsCV-3, two partitiviruses reported in cultivated radish *R. sativus*-root cv. Yidianhong, respectively, suggesting that the same or similar viruses infected different host species.

Three partitivirus-like sequences were identified in the cDNA libraries of plant samples infected by fungi. These sequences were either from fungal viruses or plant viruses. In addition, since mycoviruses are commonly found in different species of endophytic fungi of grasses [27], we cannot rule out the possibility that some virus-like sequences found in cDNA libraries of plants could actually be derived from viruses of endophytic fungi. In fact, it is yet to be determined if some of the characterized dsRNA viruses from plants are true plant or fungal viruses [13], [28].

### Identification of novel toti-, chrys- and endornavirus-like sequences

Eight virus-like sequences that were distinctly related to members of the family *Totiviridae* were discovered from cDNA libraries of plant, rust fungi, arthropods and diatoms (Table 2 and Data S1). The CP-like sequence in *Tamarix androssowii* was most closely related to that of black raspberry virus F, a toti-like virus whose sequence is publicly available only in the database. All of the totivirus-like sequences shared only low sequence identity (<50%) with known totiviruses, suggesting that they may represent the genomes of novel totivirus-like species.

**Table 2:** Totivirus, chrysovirus, endornavirus and Southern tomato virus (STV)-like sequences discovered from cDNA libraries of eukaryotic organisms.

Organism group	Organisma)	No. of totiviral RdRp		No. of totiviral CP	
		Contigb)	Singleton	Contigb)	Singleton
Diatoms	*Phaeodactylum tricornutum* (diatom)		1		
Fungi	*Puccinia striiformis f. sp.* Tritici (wheat stripe rust)		1		
	*Uromyces appendiculatus* (Bean rust)		2		
Animals	*Lepeophtheirus salmonis* (salmon louse)		1		
	*Nilaparvata lugens* (brown planthopper)		1		
	*Epiphyas postvittana* (light brown apple moth)		1		
Plants	*Tamarix androssowii*				1

Organism group	Organism	No. of STV RdRp		No. of STV CP	
		Contigb)	Singleton	Contigb)	Singleton
Plants	*Festuca arundinacea* (tall fescue)	3			
	*Festuca pratensis* (meadow ryegrass)	3	1		
	*Lolium multiflorum* (Italian ryegrass)				1
	*Triphysaria pusilla* (dwarf owl's clover)			1	
	*Zingiber officinale* (ginger)				1
	*Allium cepa* (onion)	1	2		
	*Brassica rapa subsp. pekinensis* (Chinese cabbage)		1		
Fungi	*Antonospora locustae* (microsporidians)	1			

Organism group	Organism	No. of chrysoviral RdRp		No. of chrysoviral p3	
		Contigb)	Singleton	Contigb)	Singleton
Plants	*Artemisia annua* (Sweet Wormwood)			1	
	*Zinnia violacea* (garden zinnia)		1	1	

Organism group	Organism	No. of endornaviral polyprotein	
		Contigb)	Singleton
Animals	*Caligus rogercresseyi* (Sea lice)		1
Protozoans	*Ichthyophthirius multifiliis* (white spot)	1	
Plants	Picea glauca (white spruce)	1	

a) Virus-like ESTs were detected from the cDNA libraries of these organisms. Note the possibility that certain viral sequences are possible not from the annotated host organisms.

b) Note the possibility that virus ESTs used to generate a contig are possible not from the same virus.

Fourteen virus-like sequences from plant cDNA libraries were closely related to Southern tomato virus (STV) and three other related unclassified viruses isolated from plants [19], [20], [21], with genome organizations similar to totiviruses. In addition, an RdRp-like sequence from microsporidian (*Antonospora locustae*) also was related to these four plant viruses (Table 2 and Data S1).

One chrysovirus RdRp-like and two p3 protein-like sequences were found in cDNA libraries of sweet wormwood (*Artemisia annua*) and garden zinnia (*Zinnia violacea*) (Table 2 and Data S1). These three sequences were distantly related to known chrysoviruses and each of them was most closely related to different viruses, suggesting that they may be derived from three distinct, novel viruses.

We also identified three virus-like sequences that were distantly related to the polyprotein of members of the family *Endornaviridae* from cDNA libraries of animals, protozoans, and plants (Table 2 and Data S1).

### Phylogenetic analysis of partitivirus-like sequences

**Figure 2:**
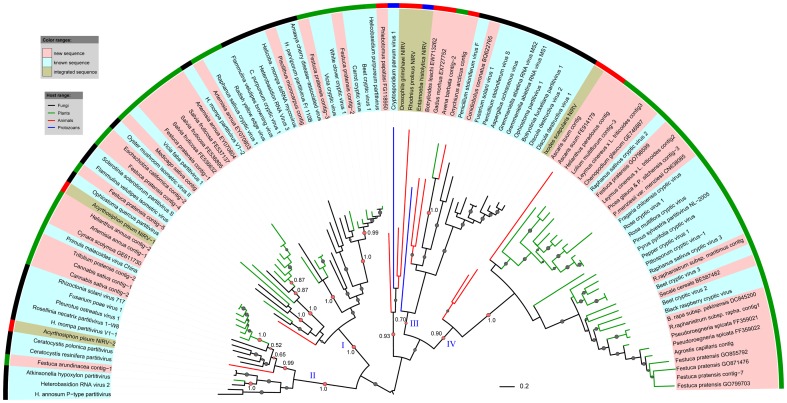
Maximum likelihood tree of partitiviral RdRp-like sequences. The phylogenetic tree were built using PhyML-mixtures based on a multiple sequence alignment generated using COBALT with the Word Size parameter setting to 3. The base tree was drawn using Interactive Tree Of Life Version 2.1.1 (http://itol.embl.de/). The tree is midpoint rooted. The p-values of approximate likelihood ratios (SH-aLRT) plotted as circle marks on the branches (only p-values>0.5 are indicated) and circle size is proportional to the the p-values. The p-values for the branches relevant to our conclusions are shown. Scale bars correspond to 0.2 amino acid substitutions per site.

**Figure 3:**
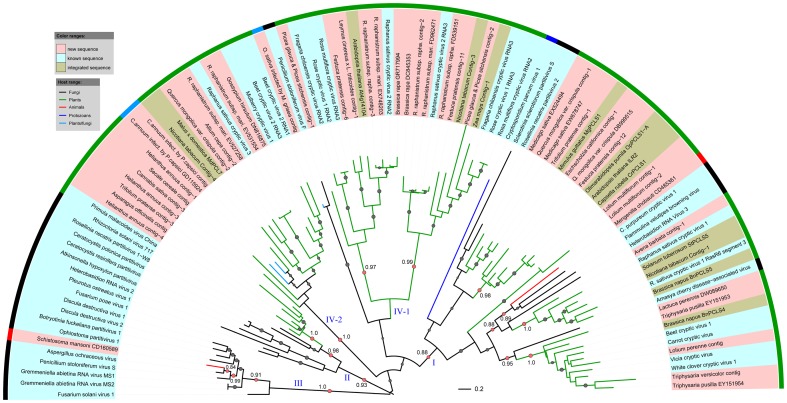
Maximum likelihood tree of partitiviral CP-like sequences. The phylogenetic tree were built using PhyML-mixtures based on a multiple sequence alignments generated using COBALT with the Word Size parameter setting to 3 and Constraint E-value parameter setting to 0.1. The base tree was drawn using Interactive Tree Of Life Version 2.1.1 (http://itol.embl.de/). The tree is midpoint rooted. The p-values of approximate likelihood ratios (SH-aLRT) plotted as circle marks on the branches (only p-values>0.5 are indicated) and circle size is proportional to the p-values. The p-values for the branches relevant to our conclusions are shown. Scale bars correspond to 0.2 amino acid substitutions per site.

To evaluate the phylogenetic relationships of the partitivirus-like sequences identified by *in silico* cloning with known viruses, we constructed maximum likelihood phylogenetic trees with amino acid sequences of RdRp or CP protein sequences. We also added recently reported endogenous partitivirus-like sequences in the phylogenetic analysis [29], [30]. As shown in Fig. 2, the RdRp tree mainly was divided into four large clades: I–IV. The CP tree has similar clades except that the CPs of viruses in clade IV of the RdRp tree formed two distinct clusters: IV-1 and IV-2 (Fig. 3). The virus-like sequences discovered here were distributed within the sub-clades of RdRp and CP trees, strongly suggesting that they were derived from members of the *Partitiviridae* family. These new viral sequences nearly doubled the amount of partitiviral sequences currently available in the public database, remarkably expanding the known diversity of partitiviruses.

The partitivirus-like sequences derived from cDNA libraries of fungi were most closely related to known fungal viruses (Fig. 2). The sequences discovered from cDNA libraries of animals Sandfly (*Phlebotomus papatasi*) and pig roundworm (*Ascaris suum*) were most closely related to integrated viral sequences in animal genomes (Fig. 2). Likewise, most of the new partitivirus-like sequences from cDNA libraries of plant samples clustered with reported plant viruses and/or integrated viruses in plant genomes. The plant virus-like sequences were generally most similar to each other and formed distinct clusters (Fig. 2 and 3). A more reasonable explanation for these results is that these partitivirus-like sequences were indeed derived from the current annotated organisms, although it is also possible that a trace fungal entophytes in the tissues of annotated organism might be the true source for certain sequences.

Clade I and II mainly consisted of mixtures of partitivirus-like sequences from plants and fungi. The mosaic distribution of plant and fungal viruses indicates the possibility of viral transmission between plants and fungi. Moreover, many of the plant viral clusters were composed of viruses from different plant families or classes (monocots or eudicots) but their phylogenies seem not to be topologically congruent with that of their hosts, suggesting that these plant partitiviruses may not be ancient origin. Considering that the branches of fungal viruses were generally locating at the base of plant viral clades (Fig. 2 and 3), it is likely that these plant partitiviruses evolved from viruses of fungi by interkingdom host jumping.

Clade IV was mainly composed of plant partitivirus-like sequences while clade IV was mainly composed of fungal partitivirus-like sequences (Fig. 2 and 3). Some of animal viral RdRp-like sequences were distantly related to those from plants and fungi and their branches were clustering deeply within each clade (Fig. 2). Furthermore, four partitivirus-like sequences from animals and protozoans clustered together and formed an extra small clade branching deeply in the RdRp tree (Fig. 2). The point of divergence of plant, fungi and animal virus-like sequences correspond to deep branching in the phylogenetic tree, implying that virus-host co-evolution seems to be possible although it is impossible to link them to any specific time scale. However, RdRp-like sequences from cDNA libraries of Tullberg (*Onychiurus arcticus*) and slender oat (*Avena barbata*) clustered together in clade III (Fig. 2). Likewise, the CP-like sequences from cDNA libraries of flatworm (*Schistosoma mansoni*) and twisted-wing parasite (*Mengenilla chobauti*) were most closely related to those of certain fungal viruses in clade I and III (Fig. 3). If these sequences were indeed derived from viruses infecting the annotated hosts, these results clearly suggest that horizontal transmission of these viruses occurred between animals and fungi or between animals and plants.

### Phylogenetic analysis of toti- and chrysovirus-like sequences

**Figure 4:**
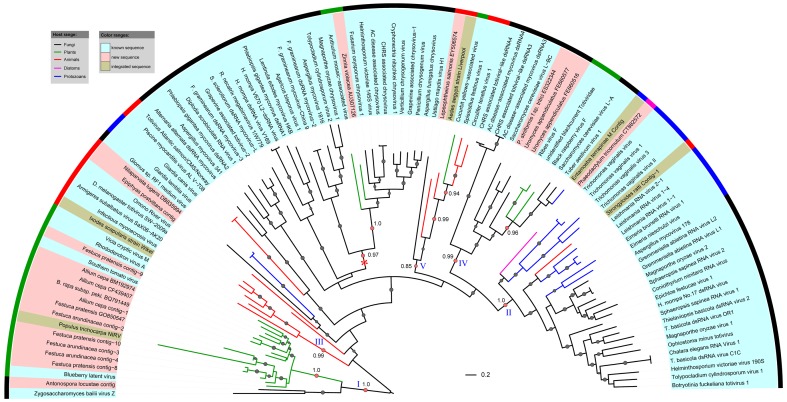
Maximum likelihood tree of totiviral, chrysoviral, and STV RdRp-like sequences. The phylogenetic tree was built using PhyML-mixtures based on a multiple sequence alignment generated using COBALT with the Word Size parameter setting to 3. The base tree was drawn using Interactive Tree Of Life Version 2.1.1 (http://itol.embl.de/). The totivirus-like group represented by Southern tomato virus (STV) was chosen as outgroup for presentation purposes. The p-values of approximate likelihood ratios (SH-aLRT) plotted as circle marks on the branches (only p-values>0.5 are indicated) and circle size is proportional to the p-values. The p-values for the branches relevant to our conclusions are shown. Scale bars correspond to 0.2 amino acid substitutions per site. Asterisk (*) indicates the chrysoviral clade.

We constructed phylogenetic tree for the toti- and chrysovirus-like sequences discovered here with all available RdRps from members of the families *Chrysoviridae* and *Totiviridae* as well as other totivirus-related unclassified viruses. Recently identified endogenous totivirus-like sequences [29] were also included in this analysis. We found that toti- and chrysovirus-like sequences actually comprise diverse viral lineages (Fig. 4). Most of the newly identified virus-like sequences were placed within the sub-clades of phylogenetic tree strongly suggesting that they were derived from members of these viral families. All STV-like sequences from plants and fungi clustered together and constituted a distinct clade (clade I). The two fungal STV-like sequences located at the base of this clade, which was more distantly related to plant sequences, suggesting that these viruses may have co-evolved with their host (fungi and plants). Similarly, the deep branching of virus-like sequence from diatoms, protozoans and fungi in clade II and these from animals in clade III is also likely to be the result of coevolution between viruses and hosts. However, it is difficult to determine how deep the co-evolution is. The three plant viruses in clade IV, however, were possibly originated from fungal viruses via interkingdom host jumping, because their branches located within cluster of fungal viruses. Likewise, the unclassified Cucurbit yellow-associated virus may have evolved from insect viruses of clade V (Fig. 4), if this virus is indeed a plant virus.

The chrysovirus-like sequences clustered together and formed a distinct clade in the phylogenetic tree (Fig. 4). The only two sequences from plants branching at the base of one sub-clade, possible represent the co-evolved viral lineage in plants.

### Phylogenetic analysis of endornavirus-like sequences

**Figure 5:**
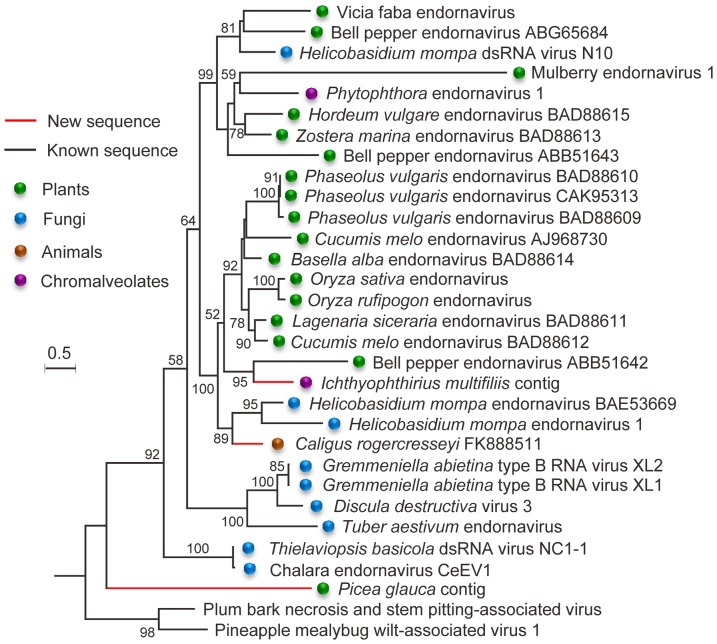
Maximum likelihood tree of polyproteins of endornavirus-like sequences. The phylogenetic tree was built using PhyML-mixtures based on a multiple sequence alignment generated using COBALT with the default parameter setting. The tree was rooted with closteroviruses *Pineapple mealybug wilt-associated virus 1* and *Plum bark necrosis and stem pitting-associated virus*, which belong to the “alpha-like” supergroup of ss(+)RNA viruses. Only p values for the approximate likelihood ratios (SH test) of >0.5 (50%) are indicated. Scale bars correspond to 0.5 amino acid substitution per site.

Phylogenetic analysis of endornavirus-like sequences shown that the plant viral sequences were generally clustered together and fungal viral sequences were generally branched at the base of plant viral clusters (Fig. 5). This is likely to be the results of coevolution of viruses with their hosts. However, horizontal transmission of viruses may have occurred between plants and fungi or chromalveolates as the fungal virus *Helicobasidium mompa* dsRNA virus N10 and two chromalveolate viruses: *Phytophthora* endornavirus 1 and *Ichthyophthirius multifiliis* contig were most closely related to certain plant viruses, respectively (Fig. 5). The virus-like sequence from Sea lice (*Caligus rogercresseyi*) clustered with those of two fungal endornaviruses of *Helicobasidium mompa* and thereby possibly evolved from fungal viruses. The virus-like sequence from white spruce (*Picea glauca*) was distantly related to other endornavirus-like sequences, possibly representing a new dsRNA viral lineage related to endornaviruses.

### The potential host range of dsRNA viruses

The dsRNA virus-like sequences discovered here are either from integrated viral sequences or infecting viruses. Although it is not certain whether all of the virus-like sequences are indeed derived from the annotated host organisms, they may indicate the potential hosts for these viruses and extend the possible host range of viruses.

**Figure 6:**
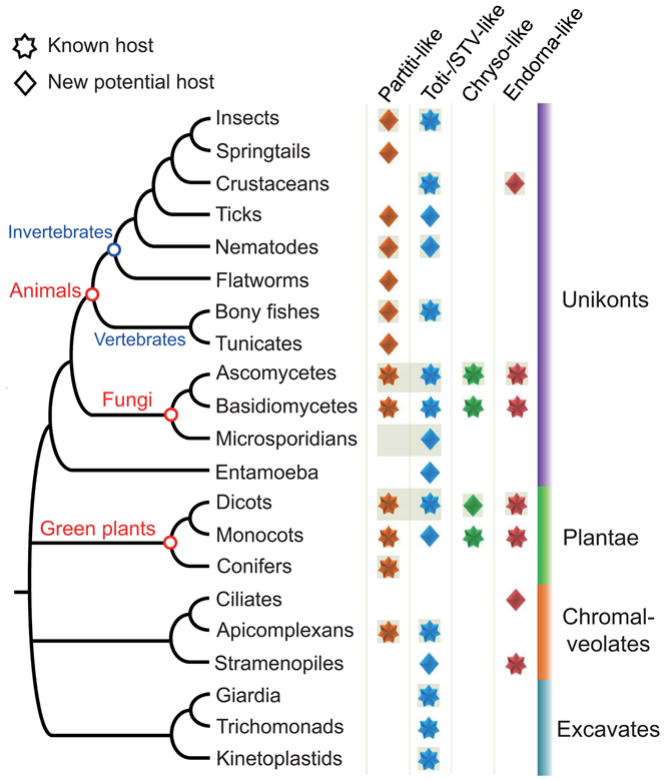
A tree of eukaryotes showing known host range of partitvirus-, totivirus-, chrysovirus- and endornavirus-like viruses. This tree was drawn base on The Tree of Life Web Project (http://tolweb.org/).

Members of the family *Partitiviridae* commonly occur in plants and fungi. To date, only one member of this family, the *Cryptosporidium parvum virus 1*, was found to infect apicomplexan protozoa of genus *Cryptosporidium*. The discovery of partitivirus-like sequences from animal cDNA libraries in this study together with our previous finding of endogenous partitiviral sequences in arthropod genomes [29] clearly suggest that these viruses can also infect animals (Fig. 6).

Totiviruses are known to infect fungi and protozoa and recently found in arthropods [11], [31], [32], [33] and fish [34]. In addition, three totiviruses and four STV-like viruses from plants have been published [19], [20], [21], [35] or are publicly available in the database. Our study further extended the possible host range of these viruses to other eukaryotic lineages (Fig. 6).

Though the only known hosts of viruses in the family *Chrysoviridae* are fungi, Anthurium mosaic-associated virus, a chryso-like virus infecting monocots is available in the database. Furthermore, we discovered three chryso-like sequences from two dicot plant species, suggesting that plants also are possible hosts of chrysoviruses.

Endornaviruses were originally discovered in plants and later found in fungi and Stramenopiles [18]. Our finding further extends the potential host range of these viruses to animals (Fig. 6).

Altogether, totiviruses and STV-like viruses have the broadest host range, including four of the five supergroups of eukaryotes: Unikonta, Plantae, Chromalveolata and Excavata [36]. However, chrysoviruses were only distributed in the former two supergroups. The potential host range of partitiviruses and endornaviruses includes three eukaryotic supergroups: Unikonta, Plantae, Chromalveolata. With the discovery of more and more viruses, the host range of these viruses will be further extended.

### Interaction and evolution of dsRNA viruses with their hosts

The partiti-, toti-, chryso-, and endornaviruses have similar features that they are transmitted vertically via spores and do not have extracellular routes for infection and have little or no effects on their hosts [12], [13]. These characteristics indicate that these viruses may have co-evolved with their hosts [24], [25], but there is no clear phylogenetic evidence supporting this. These viruses were initially found in fungi or plants but shown in recent reports and this study to be widely distributed in eukaryotic supergroups. The origins of *Partitiviridae* and *Totiviridae* have been revealed to be ancient and antedate the radiation of eukaryotic supergroups [26]. Our phylogenetic analysis showed that some lineages of these dsRNA families from different host supergroups are branching deeply in the phylogenetic tree. The phylogenetic pattern indicates that these viral lineages may have co-evolved with their host supergroups. However, it is difficult to determine how deep the co-evolution is. The extent of co-evolution could either be complete co-evolution that virus split corresponding to the host split or partial co-evolution that ancestral viruses transmitted between different host supergroups followed by co-diverged with respective hosts. Our data also revealed that recent horizontal transmission of these viruses may have occurred between different host supergroups (such as fungi and plants). Although interkingdom host jumping is not occurred easily because it would require entry into the germline, there is sufficient opportunity to occur during the long evolutionary history with their hosts.

### A simple, effective approach for discovery of novel viruses

In this study, by using the in *silico* cloning approach, we discovered numerous novel virus-like sequences from NCBI EST database, representing members of families *Partitiviridae*, *Totiviridae, Endornaviridae*, and *Chrysoviridae*. The new sequences identified in this study are either from novel viruses or from known but yet unsequenced viruses. These viral sequences provide extended the potential host range of related viruses and help to shed light on their origin and evolution.

We failed to find EST sequences similar to three other dsRNA families, *Birnaviridae*, *Picobirnaviridae* and *Reoviridae*. It is possible that samples for cDNA library construction containing viruses are easily detected due to the fact that these viruses generally cause serious disease on their hosts.

This method we used seemed to be more effective for identification of plant viruses. Considering the extensive diversity of plant viruses and the rapid increase of plant EST sequencing, the *in silico* cloning approach may has a broad application prospect in plant virology. In fact, similar approaches were used to identify several families of plant positive-sense ssRNA viruses. Numerous EST sequences similar to these viruses were also found. Many of them may belong to new species or genera. We also found many plant virus-like sequences from cDNA libraries of insects and nematodes, suggesting new viral vectors or new host range for these plant viral lineages (Liu Huiquan *et al.*, unpublished data).

### Conclusions

In this study, we demonstrated the application of the *in silico* cloning approach for discovery of novel dsRNA viral sequences. By using this method, we obtained 91 partitivirus-like, 22 toti- or STV-like, 3 chrysovirus-like and 3 endornavirus-like novel sequences. Some of these virus-like sequences were discovered from eukaryotic lineages which are not known to be hosts for these viruses. Furthermore, phylogenetic analysis of these new virus-like sequences with those of known dsRNA viruses revealed that these viruses may have co-evolved with respective host supergroups over a long evolutionary time frame while potential viral horizontal transmission was also likely to be occurred between different host supergroups. The phylogenetic analysis also revealed that some of plant partitiviruses may have originated from mycoviruses by interkingdom host jumping. Our findings extend the diversity and possible host range of dsRNA viruses and offers insight into the origin and evolution of relevant viruses with their hosts.

## Materials and Methods

### DsRNA viruses cloning in *silico*

We firstly selected and downloaded the protein sequences derived from representative viruses in each genus of dsRNA families, *Birnaviridae*, *Picobirnaviridae*, *Reoviridae Endornaviridae*, *Chrysoviridae*, *Partitiviridae*, and *Totiviridae* from viral genome databases at the NCBI website (http://www.ncbi.nlm.nih.gov/genomes/GenomesHome.cgi?taxid=10239). These viral sequences were then used as seed queries to search against the NCBI EST database by Netblast (blastcl3) program with tBLASTn strategy. All non-redundant matches from these searches with E-values≤1e-5 were extracted and were divided into different groups according to different sources of species. The ESTs in each group were used to construct contigs with CAP3 sequence assembly program where overlapping regions of EST sequences show at least 97% sequence identity. The resulting contigs and singletons were used as BLASTx queries against the non-redundant (NR) protein database to confirm the assembly quality and the relationships between these and the known viruses. If the contigs and singletons from one species have high sequence identity (95% DNA) with the reported viruses in the same species, these sequences were discarded and not analyses further. The analyses were completed by March 2011.

### Sequence alignment and phylogenetic analysis

The software package DNAMAN 7 (Lynnon Biosoft, USA) was used for sequence annotations, including nucleotide statistics and ORF searching. The putative peptides of viral contigs and singletons were obtained according to BLASTx hits and ORF predictions were checked manually. Multiple alignments of protein sequences were constructed using COBALT [37] and manually edited. To give the best alignment, the alignment parameter Constraint E-value and Word Size were adjusted for different datasets.

Maximum likelihood (ML) phylogenies were estimated using amino acid sequence alignments with PhyML-mixtures [38], [39], assuming the EX2 mixture model [39] and SPR tree topologies search strategy. Gaps in alignment are systematically treated as unknown characters. The reliability of internal branches was evaluated based on SH-like approximate likelihood ratio test (SH-aLRT) statistics.

## Supporting Information

Data S1 **Summary of virus-like EST sequences obtained by cloning** ***in silico*** **.**
(XLS)

## References

[1] Balijja A, Kvarnheden A, Turchetti T (2008) A non-phenol-chloroform extraction of double-stranded RNA from plant and fungal tissues. J Virol Methods 152: 32–37. 18598720 10.1016/j.jviromet.2008.06.001

[2] Froussard P (1992) A random-PCR method (rPCR) to construct whole cDNA library from low amounts of RNA. Nucleic Acids Res 20: 2900. 1614887 10.1093/nar/20.11.2900 PMC336952

[3] Delwart EL (2007) Viral metagenomics. Rev Med Virol 17: 115–131. 17295196 10.1002/rmv.532 PMC7169062

[4] Edwards RA, Rohwer F (2005) Viral metagenomics. Nat Rev Microbiol 3: 504–510. 15886693 10.1038/nrmicro1163

[5] Cox-Foster DL, Conlan S, Holmes EC, Palacios G, Evans JD, et al (2007) A metagenomic survey of microbes in honey bee colony collapse disorder. Science 318: 283–287. 17823314 10.1126/science.1146498

[6] Culley AI, Lang AS, Suttle CA (2006) Metagenomic analysis of coastal RNA virus communities. Science 312: 1795–1798. 16794078 10.1126/science.1127404

[7] Kim KH, Chang HW, Nam YD, Roh SW, Kim MS, et al (2008) Amplification of uncultured single-stranded DNA viruses from rice paddy soil. Appl Environ Microbiol 74: 5975–5985. 18708511 10.1128/AEM.01275-08 PMC2565953

[8] Victoria JG, Kapoor A, Dupuis K, Schnurr DP, Delwart EL (2008) Rapid identification of known and new RNA viruses from animal tissues. PLoS Pathog 4: e1000163. 18818738 10.1371/journal.ppat.1000163 PMC2533695

[9] Kreuze JF, Perez A, Untiveros M, Quispe D, Fuentes S, et al (2009) Complete viral genome sequence and discovery of novel viruses by deep sequencing of small RNAs: a generic method for diagnosis, discovery and sequencing of viruses. Virology 388: 1–7. 19394993 10.1016/j.virol.2009.03.024

[10] Vodovar N, Goic B, Blanc H, Saleh MC (2011) In silico reconstruction of viral genomes from small RNAs improves virus-derived small interfering RNA profiling. J Virol 85: 11016–11021. 21880776 10.1128/JVI.05647-11 PMC3194930

[11] Wu Q, Luo Y, Lu R, Lau N, Lai EC, et al (2010) Virus discovery by deep sequencing and assembly of virus-derived small silencing RNAs. Proc Natl Acad Sci U S A 107: 1606–1611. 20080648 10.1073/pnas.0911353107 PMC2824396

[12] King AMQ, Adams MJ, Carstens EB, Lefkowitz EJ, editors. Virus Taxonomy: Ninth Report of the International Committee on Taxonomy of Viruses. New York: Elsevier.

[13] Ghabrial SA, Suzuki N (2009) Viruses of Plant Pathogenic Fungi. Annual Review of Phytopathology 47: 353–384. 10.1146/annurev-phyto-080508-081932 19400634

[14] Blawid R, Stephan D, Maiss E (2008) Alphacryptovirus and Betacryptovirus. In: Encyclopedia of virology 3rd ed, vol 1. Mahy BWJ, Van Regenmortel MHV, editors. Oxford: Elsevier. 98–104.

[15] Ghabrial SA, Ochoa WF, Baker TS, Nibert ML (2008) Partitiviruses: General Features. In: Encyclopedia of virology 3rd ed, vol 4. Mahy BWJ, Van Regenmortel MHV, editors. Oxford: Elsevier. 68–75.

[16] Tavantzis S (2008) Partitiviruses of Fungi. In: Encyclopedia of virology 3rd ed, vol 4. Mahy BWJ, Van Regenmortel MHV, editors. Oxford: Elsevier. 63–68.

[17] Ghabrial SA (2008) Chrysoviruses. In: Encyclopedia of virology 3rd ed, vol 1. Mahy BWJ, Van Regenmortel MHV, editors. Oxford: Elsevier. 503–513.

[18] Fukuhara T, Moriyama H (2008) Endornavirus. In: Encyclopedia of virology 3rd ed, vol 2. Mahy BWJ, Van Regenmortel MHV, editors. Oxford: Elsevier. 109–116.

[19] Martin RR, Zhou J, Tzanetakis IE (2011) Blueberry latent virus: An amalgam of the Partitiviridae and Totiviridae. Virus Research 155: 175–180. 20888379 10.1016/j.virusres.2010.09.020

[20] Sabanadzovic S, Abou Ghanem-Sabanadzovic N, Valverde RA (2010) A novel monopartite dsRNA virus from rhododendron. Arch Virol 155: 1859–1863. 20721591 10.1007/s00705-010-0770-5

[21] Sabanadzovic S, Valverde RA, Brown JK, Martin RR, Tzanetakis IE (2009) Southern tomato virus: The link between the families Totiviridae and Partitiviridae. Virus Research 140: 130–137. 19118586 10.1016/j.virusres.2008.11.018

[22] Chiba S, Salaipeth L, Lin YH, Sasaki A, Kanematsu S, et al (2009) A Novel Bipartite Double-Stranded RNA Mycovirus from the White Root Rot Fungus Rosellinia necatrix: Molecular and Biological Characterization, Taxonomic Considerations, and Potential for Biological Control. Journal of Virology 83: 12801–12812. 19828620 10.1128/JVI.01830-09 PMC2786845

[23] Lin YH, Chiba S, Tani A, Kondo H, Sasaki A, et al (2012) A novel quadripartite dsRNA virus isolated from a phytopathogenic filamentous fungus, Rosellinia necatrix. Virology 426: 42–50. 22321722 10.1016/j.virol.2012.01.013

[24] Ghabrial SA (1998) Origin, adaptation and evolutionary pathways of fungal viruses. Virus Genes 16: 119–131. 9562896 10.1023/A:1007966229595 PMC7089520

[25] Villarreal LP (2005) Viruses and the evolution of life. Washington, DC: American Society for Microbiology.

[26] Koonin EV, Wolf YI, Nagasaki K, Dolja VV (2008) The Big Bang of picorna-like virus evolution antedates the radiation of eukaryotic supergroups. Nat Rev Microbiol 6: 925–939. 18997823 10.1038/nrmicro2030

[27] Herrero N, Sanchez Marquez S, Zabalgogeazcoa I (2009) Mycoviruses are common among different species of endophytic fungi of grasses. Arch Virol 154: 327–330. 19125219 10.1007/s00705-008-0293-5

[28] Salem NM, Golino DA, Falk BW, Rowhani A (2008) Complete nucleotide sequences and genome characterization of a novel double-stranded RNA virus infecting Rosa multiflora. Arch Virol 153: 455–462. 18172568 10.1007/s00705-007-0008-3

[29] Liu H, Fu Y, Jiang D, Li G, Xie J, et al (2010) Widespread horizontal gene transfer from double-stranded RNA viruses to eukaryotic nuclear genomes. J Virol 84: 11876–11887. 20810725 10.1128/JVI.00955-10 PMC2977895

[30] Chiba S, Kondo H, Tani A, Saisho D, Sakamoto W, et al (2011) Widespread endogenization of genome sequences of non-retroviral RNA viruses into plant genomes. PLoS Pathog 7: e1002146. 21779172 10.1371/journal.ppat.1002146 PMC3136472

[31] Poulos BT, Tang KF, Pantoja CR, Bonami JR, Lightner DV (2006) Purification and characterization of infectious myonecrosis virus of penaeid shrimp. J Gen Virol 87: 987–996. 16528049 10.1099/vir.0.81127-0

[32] Zhai Y, Attoui H, Mohd Jaafar F, Wang HQ, Cao YX, et al (2010) Isolation and full-length sequence analysis of Armigeres subalbatus totivirus, the first totivirus isolate from mosquitoes representing a proposed novel genus (Artivirus) of the family Totiviridae. J Gen Virol 91: 2836–2845. 20702653 10.1099/vir.0.024794-0

[33] Spear A, Sisterson MS, Yokomi R, Stenger DC (2010) Plant-feeding insects harbor double-stranded RNA viruses encoding a novel proline-alanine rich protein and a polymerase distantly related to that of fungal viruses. Virology 404: 304–311. 20541786 10.1016/j.virol.2010.05.015

[34] Haugland O, Mikalsen AB, Nilsen P, Lindmo K, Thu BJ, et al (2011) Cardiomyopathy syndrome of atlantic salmon (Salmo salar L.) is caused by a double-stranded RNA virus of the Totiviridae family. J Virol 85: 5275–5286. 21411528 10.1128/JVI.02154-10 PMC3094960

[35] Cox S, Mayo M, Jones AT (2000) The occurrence of dsRNA species in apparently healthy and virus-infected Ribes cultivars, and evidence that one such species originates from a member of the virus family Totiviridae. European journal of plant pathology 106: 353–364.

[36] Keeling PJ, Burger G, Durnford DG, Lang BF, Lee RW, et al (2005) The tree of eukaryotes. Trends Ecol Evol 20: 670–676. 16701456 10.1016/j.tree.2005.09.005

[37] Papadopoulos JS, Agarwala R (2007) COBALT: constraint-based alignment tool for multiple protein sequences. Bioinformatics 23: 1073–1079. 17332019 10.1093/bioinformatics/btm076

[38] Guindon S, Gascuel O (2003) A simple, fast, and accurate algorithm to estimate large phylogenies by maximum likelihood. Syst Biol 52: 696–704. 14530136 10.1080/10635150390235520

[39] Le SQ, Lartillot N, Gascuel O (2008) Phylogenetic mixture models for proteins. Philos Trans R Soc Lond B Biol Sci 363: 3965–3976. 18852096 10.1098/rstb.2008.0180 PMC2607422

